# *Acacia hydaspica* R. Parker prevents doxorubicin-induced cardiac injury by attenuation of oxidative stress and structural Cardiomyocyte alterations in rats

**DOI:** 10.1186/s12906-017-2061-0

**Published:** 2017-12-29

**Authors:** Tayyaba Afsar, Suhail Razak, Khalid Mujasam Batoo, Muhammad Rashid Khan

**Affiliations:** 1 Department of Biochemistry, Faculty of Biological Sciences, Quaid-i-Azam University, Islamabad, Pakistan; 2 Department of Animal Sciences, Faculty of Biological Sciences, Quaid-i-Azam University, Islamabad, Pakistan; 3 Department of Community Health Sciences, College of Applied Medical Sciences, King Saud University, Riyadh, Kingdom of Saudi Arabia; 4 King Abdullah Institute for Nanotechnology, King Saud University, P.O.Box 2455, Riyadh, 11451 Kingdom of Saudi Arabia

**Keywords:** Doxorubicin, Cardiotoxicity, Oxidative stress, Cardiac function biomarkers, Antioxidants, Polyphenolics

## Abstract

**Background:**

The use of doxorubicin (DOX) an anthracycline antineoplastic agent is withdrawn due to its cardio-toxic side effects. Oxidative stress has been recognized as the primary cause of DOX induced cardiotoxicity. We have investigated whether polyphenol rich ethyl acetate extract of *Acacia hydaspica* (AHE) can attenuate doxorubicin-induced cardiotoxicity via inhibition of oxidative stress.

**Methods:**

AHE was administered orally to rats once daily for 6 weeks at doses of 200 and 400 mg/kg b.w. DOX (3 mg/kg b.w. i.p., single dose/week) was administered for 6 weeks (chronic model). The parameters studied to evaluate cardioprotective potential were the serum cardiac function biomarkers (CK, CKMB, AST and LDH), hematological parameters, cardiac tissue antioxidant enzymatic status and oxidative stress markers, and histopathological analysis to validate biochemical findings.

**Results:**

Chronic 6 week treatment of DOX significantly deteriorated cardiac function biomarkers and decreased the activities of antioxidant enzymes, whereas significant increase in oxidative stress biomarkers was noticed in comparison to control group. AHE dose dependently protected DOX-induced leakage of cardiac enzymes in serum and ameliorated DOX-induced oxidative stress; as evidenced by decreasing lipid peroxidation, H_2_O_2_ and NO content with increase in phase I and phase II antioxidant enzymes. Doxorubicin treatment produced severe morphological lesions, leucopenia, decrease in red blood cell counts and hemoglobin concentrations. AHE co-treatment protected the heart and blood elements from the toxic effects of doxorubicin as indicated by the recovery of hematological parameters to normal values and prevention of myocardial injuries in a dose dependent way. The protective potency of AHE (400 mg/kg b.w) was equivalent to silymarin.

**Conclusion:**

Results revealed that AHE showed protective effects against DOX induce cardiotoxicity. The protective effect might attribute to its polyphenolic constituents and antioxidant properties. AHE might be helpful in combination therapies as safer and efficient.

## Background

Cardiovascular disease (CVD) is the second leading cause of long-term morbidity and mortality among cancer survivors. Conventional chemotherapy and targeted therapies are associated with an increased risk of myocardial dysfunction to irreversible heart failure or even death [1]. The anthracycline anticancer drug doxorubicin (DOX) also recognized as adriamycin, is an effective and frequently used chemotherapeutic agent for various malignancies [2–6]. Regardless of its great antitumor efficiency, its use in chemotherapy is limited due to its varied side effects. Its most prevalent and unavoidable side effect is cardiotoxicity [5, 7, 8]. DOX administration outcomes in permanent cardiomyopathy even lapse of years after the completion of chemotherapy [9–11]. Even when taking into account lower cumulative doses and cardioprotective regimens now in use, DOX cardiotoxicity still occurs, and its prevention and management remains of concern to both cardiologists and oncologists. There appears to be multifactorial patho-mechanisms behind DOX-associated late cardiotoxicity, but predominantly oxidative stress is linked to redox cycling of the drug due to the overproduction of superoxide radicals (O2 *−* ***·***); which is the source for generating hydrogen peroxide and much more toxic hydroxyl radical and hence inducing oxidative stress [12–14]. Most the reactions involved in the DOX radical generation were catalyzed in the liver instead of heart, but due to relatively low antioxidant defense of cardiomyocytes makes the heart a most prominent target for DOX toxicity and the extent of the doxorubicin-persuaded oxidative trauma is up to 10 times higher in the cardiac tissue as compared to other tissues (liver, kidney, spleen). Structural cardiomyocyte alterations and cell death induced by DOX is mediated in part by reactive oxygen species (ROS) generated in iron-dependent chemical reactions. ROS lead to the peroxidation of myocyte membranes and, after calcium influx, into the intracellular space, which can ultimately lead to permanent myocyte damage [15].

Over the years, researchers have testified that plants containing phenolics and flavonoids exhibit a large array of biological activities including cardio-protection, anti-fibrosis and anticancer [16, 17]. Cardiotoxicity associated with DOX treatment has been successfully prevented by different medicinal plants with antioxidant activity. Thus, it is well justified to explore more plant derived-natural compounds that inhibit the cardiotoxicity of DOX and improve its chemotherapeutic efficiency [18, 19].

*Acacia s*pecies are rich sources of polyphenolic compounds, known to have strong antioxidant properties that help in the prevention of various oxidative stress related diseases including cardiovascular, neurodegenerative and cancer [20–22]. *Acacia hydaspica* R. Parker; synonym *A. eburnea* belongs to family Leguminosae [23], possesses antioxidant, anticancer, anti-hemolytic, anti-inflammatory, antipyretic, analgesic and antidepressant potentials. These activities might attribute to the presence of various active secondary metabolites i.e. gallic acid, catechin, rutin, caffeic acid, 7-*O*-galloyl catechin, +catechin and methyl gallate [24–26]. Polyphenolic compounds isolated from *A. hydaspica* induce apoptosis and inhibit various pro-survival signaling pathways in breast and prostate cancer cell lines, indicating their potential in molecular target based adjuvant chemotherapy [27]. Ethyl acetate extract of *A. hydaspica* (AHE) was selected for the current investigation due to its significant antioxidant capacity [26], and the presence of catechin and gallic acid as chief component, substantial total phenolic and flavonoid content (Table 1). Previous researches indicated that catechins possess persuasive antioxidant, anti-inflammatory, immunomodulatory, cardioprotective, and anticancer potentials. Catechin showed cardioprotective effects in rats and ameliorated electrocardiogram (ECG) changes and myocardial contractility. The underlying mechanisms involved in the cardioprotective effects of catechin could be attributed to its antioxidant and anti-apoptotic activities [28]. *Acacia* species have also been tested in animal models to evaluate their cardioprotective potential. *A. Senegal* Gum Arabic showed potential protective effects against doxorubicin-induced cardiotoxicity by reducing Dox induced cardiac tissue damages and reducing altered serum biomarkers in rats [29]. Another study in rabbits indicated that *A. senegal* seed extract administration ameliorated atherogenic diet induced cardiac LPO and histopathological abnormalities in aorta wall, heart and kidney.

**Table 1:** Extraction yield, TPC, TFC, and chemical constituents in *A. hydaspica* ethyl acetate extract (AHE)

Analysis (AHE fraction)	Observations	(References)
Extraction yield (%)	27.77%	[25]
TPC (mg gallic acid equivalent/g dry sample)	120.3 ± 1.15	[25]
TFC (mg rutin equivalent/g dry sample)	129 ± 1.32	[25]
HPLC-DAD (Identification of compounds with reference to standards)	i. Gallic acid (275 nm, RT: 4.516, 52.92 μg/100 mg dry powder)	[25]
	ii. Catechin (279 nm, RT 11.427, 8648 μg/100 mg dry powder)	
	iii. Myricetin (368 nm, RT: 17.082, 34.60 μg/100 mg dry powder)	
Purified isolated compounds (NMR characterization of compounds)	i. 7-*O*-galloyl catechin (GC)(187.5 mg/g)	[24, 27]
	ii. Catechin (C), (100 mg/g)	
	iii. Methyl gallate (MG), (37.5 mg/g)	

*TPC* Total Phenolic content, *TFC* Total flavonoid content

Information derived from previous lab investigations

Based on earlier research on the cardio-protective potential of the *Acacia* species, polyphenolic compounds in animal models and antioxidant properties of *A. hydaspica*; current investigation was done to determine the potential of ethyl-acetate extract of *A. hydaspica* to attenuate DOX-induced cardiac toxicity and oxidative stress in rats. In this regard the activity level of various antioxidant enzymes of cardiac tissues, histopathological evaluation along with biochemical serum cardiac function markers and hematological parameters were investigated to evaluate the protective potential of *A. hydaspica* against DOX induced cardiac damages.

## Methods

### Plant collection and preparation of AHE extract

Aerial parts (bark, twigs, and leaves) of *A. hydaspica* were collected from Kirpa charah area Islamabad, Pakistan. Plant specimen was identified by Dr. Sumaira Sahreen (Curator at Herbarium of Pakistan, Museum of Natural History, Islamabad). Plant specimen with Accession No. 0642531 was deposited at the Herbarium of Pakistan, Museum of Natural History, Islamabad. *A. hydaspica* methanol extract was fractionated as previously described [25], and its ethyl acetate extract (AHE) (the most bioactive extract under in vitro examinations and containing bioactive polyphenols [27]) was selected for further in vivo investigation.

### Drug and plant dose preparation

Doxorubicin (DOX) injection was obtained from Sigma-Aldrich (St. Louis, MO, U.S.A.) and dissolved in saline to make appropriate dose for administration. An entire doxorubicin dose of 18 mg/kg body weight was inoculated to rats during the experimental period [30]. Silymarin (100 mg/kg b.w) and AHE (400 and 200 mg/kg b.w) were freshly prepared in distilled water before dosing [31].

### Ethics statement

Animals were cared for in accordance with the standard guidelines of national institute of animal health (NIH guidelines) to conduct the experiment effectively. The protocol was approved by the Ethics Committee of Animal Care and Use at Quaid-i-Azam University, Islamabad (Approval No.Bch#264).

### Experimental animals

Male Sprague Dawley rats (200–225 g) were kept in the Primate Facility at Quaid-i-Azam University, Islamabad. Animals were acclimatized for 7 days to laboratory conditions. The animals were placed in conventional steel cages at room temperature, fed with standard pellet diet and tap water under 12 h light/dark cycle at 25 ± 3 °C.

### Acute toxicity evaluation

The acute toxicity study was conducted as per the guidelines 425 of Organization for Economic Cooperation and Development (OECD) for testing of chemicals for acute oral toxicity [32]. Male Sprague Dawley rats were kept in fasting conditions for overnight with just water availability. Three animals were orally administered with dose-of 50 mg/kg bw and were monitored for mortality rate for 72 h. No initial progression of toxicity was observed, but the methodology was subsequently followed with augmented amount of oral doses i.e., 100, 200, 400, 1000, 2000, 3000 and 4000 mg/kg bw of AHE, while the control group received saline (10 ml/kg). Three animals were used for each treatment. General behavioral changes were detected by the previously described procedure [33]. Animals were observed continuously for 2 h and parameters which were observed were convulsion, tremor, aggression, excitation, loss of grasp, altered reactivity to touch, and sedation [34]. AHE was found to be safe at all tested doses (up to 4000 mg/kg b.w) and it did not induced any noxious symptom in rats like sedation, convulsions, diarrhea and irritation. 200 and 400 mg/kg bw doses were selected for the evaluation of cardioprotective activity.

### Treatment regime

The study protocol was designed according to previous studies [35–37] with slight modifications. Animals were randomly divided into six groups (*n* = 6) and were subjected to following treatments.

- Group I: Control; received six doses of normal saline (0.4 ml, i.p.) for 6 weeks (one dose/week) and distilled water oraly for 6 weeks.
- Group II: DOX treated; received 3 mg/kg b.w. (i.p.) dose of DOX, one dose per week for 6 weeks (18 mg/kg b.w. total dose) for inducing organ toxicity, and oral distilled water for 6 weeks.
- Group III: AHE treated; received a single oral dosage of 400 mg/kg b.w. /day for 6 weeks.
- Group IV: DOX+ AHE 200 mg/kg; received a single oral dose of 200 mg/kg b.w. /day for 6 weeks with DOX i.p. injection once per week.
- Group V: DOX+ AHE 400 mg/kg; received one dose of 400 mg/kg b.w., p.o. /day for 6 weeks with DOX i.p. injection once per week.
- Group VI: DOX+ Silymarin; received 2 oral doses of 100 mg/kg b.w/ week (12 doses/6 weeks) with DOX i.p. injection once per week.

Initial and final body weights of rats were recorded.

### Sample preparation

Animals were euthanized by cervical dislocation and dissected from ventral side. Blood was collected and centrifuged at 10,000 rpm for 15 min at 4 °C to obtained serum. Serum samples were kept in a freezer at −80 °C for subsequent biochemical analysis. After taking blood, the heart was removed and washed in ice cold saline and weigh. Subsequently, half of the organs were treated with liquid nitrogen and stored at −80 °C for further enzymatic analysis while the other half was processed for histology.

### Cardiotoxicity indices

Different cardiac marker enzymes were used to perform the cardiac function tests such as aspartate aminotransferase (AST), creatine kinase (CK) and creatine kinase-MB (CK-MB) and lactate dehydrogenase (LDH) activities were estimated in serum samples by the standard procedure of AMP diagnostic kits (Stattogger Strasse 31b 8045 Graz, Austria).

### Hematological parameters

Heparinized blood samples were used for the evaluation of hematological parameters. Amount of red blood cells (RBCs), white blood cells (WBCs), platelets, hemoglobin concentration (Hb%), mean corpuscle volume (MCV), packed cells volume (PCV), mean corpuscle hemoglobin (MCH), hematocrit value (HTC), mean corpuscle hemoglobin concentration (MCHC), neutrophils and lymphocytes were examined following standard methods using an automated analyzer (Mindray Auto hematology Analyser BC-5500).

#### Biochemical studies of heart

##### Homogenate preparation

100 mg of heart tissue was homogenized in 10 volumes of 100 mM KH2PO4 buffer containing 1 mM EDTA, pH 7.4 and centrifuged at 12,000×g for 30 min at 4 °C. The supernatant was collected and used for the following experiments.

##### Estimation of tissue protein content

Lowry et al. procedure was followed to estimate the total soluble proteins within the tissue samples [38]. To the tissue homogenate 200 μl of 1.1 M potassium phosphate buffer-(pH 8.0) was added to dilute the tissue sample. A volume of 1 ml of alkaline copper-solution was added to-this blend, and placed at room temperature. After incubation for 20 min, 200 μl of Folin-Ciocalteu phenol reagent was added. Reaction tubes containing the test mixtures were then vortexed and incubated again at 37 °C for 20 min. At 650 nm OD was measured spectrophotometrically. Total soluble proteins of the tissue samples were then detected using standard curve of bovine serum-albumin (BSA).

#### Analysis of cardiac tissue antioxidant status

##### Catalase (CAT) activity

On the basis of decomposition of hydrogen peroxide CAT activity were analyzed by following the modified protocol of Khan et al. [39]. In brief, CAT reaction solution consists of 625 μl of 50 mM of potassium phosphate buffer (pH 5), 100 μl of 5.9 mM H_2_O_2_ and 35 μl enzyme extract, change in absorbance was noted for one minute at a wavelength of 240 nm by spectrophotometer. A change of 0.01 in absorbance for one minute was taken as one unit of CAT activity.

##### Peroxidase assay (POD)

Activities of POD were evaluated based on guaiacol peroxidation [39]. POD reaction solution was prepared by adding 75 μl hydrogen peroxide (40 mM), 25 μl guaiacol (20 mM), and 25 μl of supernatant to 625 μl of potassium phosphate buffer (pH 5.0, 50 mM) in sequence. Change in absorbance of the reaction solution at 470 nm was observed for one minute. One unit POD activity is equivalent to change in absorbance of 0.01 as units/min.

##### Superoxide dismutase (SOD) activity

Phenazine methosulphate and sodium pyrophosphate buffers were exploited for the assessment of SOD activity [40]. Centrifugation of tissue homogenate was done at 1500×g for 10 min followed by 10,000×g for 20 min. 150 μl supernatant was added to the reaction mixture containing 600 μl of 0.052 mM sodium pyrophosphate buffer (pH 7.0) and 186 mM of phenazine methosulphate (50 μl). To initiate enzymatic reaction 100 μl of 780 μM NADH was added. After 1 min, glacial acetic acid (500 μl) was added to stop the reaction. The color intensity was measured at wavelength of 560 nm. Results were expressed in units/mg protein.

##### Quinone reductase assay (QR)

The Quinone reductase activity was evaluated as described earlier [41]. 100 μl of tissue homogenate was added to 3 ml of a reaction mixture comprised of 2.13 ml Tris-HCl buffer (25 mM; pH 7.4), 700 μl of BSA, 100 μl of FAD, 20 μl of NADPH (0.1 mM). Reduction of dichlorophenolindophenol (DCPIP) was noted at 600 nm. Enzyme potency was estimated as nM of DCPIP reduced/min/mg protein using molar extinction coefficient of 2.1 × 10^4^/M/cm.

##### Reduced glutathione (GSH) estimation test

The concentration of reduced glutathione was assessed as described by Jollow [42]. The basis of this method-is based on the breakdown-of 1, 2-dithio-bis nitro-benzoic acid (DTNB) by sulfosalicylic acid, as a result yellow-color is produced. The yellow color produced was read immediately at 412 nm on a-spectrophotometer and was expressed as μM GSH/g tissue.

##### Glutathione-S-transferase (GST)

Scheme of Habig et al. [43] was followed for the estimation of GST potency. The assay principle relies on the formation of CDNB conjugate. 150 μl aliquot of tissue homogenate was added to 720 μl of sodium phosphate buffer together with 100 μl of reduced glutathione (1 mM) and 12.5 μl of CDNB (1 mM). OD was recorded at 340 nm by spectrophotometer. GST activity was estimated as amount of CDNB conjugate formed per minute per mg protein using molar coefficient of 9.61 × 10^3^/M/cm.

##### Glutathione reductase assay (GSR)

GSR activity was analyzed by following the method of Carlberg and Mannervik [44]. 100 μl of supernatant samples were amalgamated with 1.65 ml phosphate buffer: (0.1 M; pH 7.6), 100 μl EDTA (0.5 mM), 50 μl oxidized glutathione (1 mM) and 100 μl NADPH (0.1 mM). The OD was measured at 340 nm after mixing. Enzyme activity was estimated as nM NADPH oxidized/min/mg protein, using molar extinction coefficient of 6.22 × 10^3^/M/cm.

##### Glutathione peroxidase assay (GSH-Px)

Glutathione peroxidase activity was assessed as described previously [45]. 100 μl supernatant samples were mixed with 100 μl EDTA (1 mM), 1.49 ml phosphate buffer (0.1 M; pH 7.4), 100 μl sodium azide (1 m M), 50 μl glutathione reductase (1 IU/ml), 50 μl GSH (1 mM), 100 μl NADPH (0.2 mM) and 10 μl H_2_O_2_ (0.25 mM). The loss of NADPH was recorded at 340 nm at room temperature. The absorbance was noted at 340 nm, and GSH-Px activity was assessed by using a molar extinction coefficient of 6.23 × 10^3^/M/cm.

##### γ-Glutamyl transpeptidase (γ-GT)

The activity of γ-GT was checked following the scheme of Orlowski et al. [46]. Reaction solution consist of an aliquot of 50 μl tissue homogenate supernantant, 250 μl of glutamyl nitroanilide (4 mM), 250 μl of glycyl glycine (40 mM) and 250 μl of MgCl2 (11 mM) which was prepared in 185 mM Tris HCl buffer at room temperature. After 10 min of incubation, the reaction was stopped with the addition of 250 μl 25% trichloro acetic acid a and centrifuge at 2500×g for 10 min. The OD of supernatant was determined at 405 nm. Activity of γ-GT was expressed as nM nitroaniline formed per min per mg protein by using molar extinction coefficient of 1.75 × 10^3^/M/cm.

#### Oxidative stress biomarkers

##### Lipid peroxidation assay (LPO)

The analysis of lipid peroxides was done by following protocol of Iqbal and Wright [47]. The reaction mixture consists of 290 μl phosphate buffer (0.1 M, pH 7.4), 10 μl ferric chloride (100 mM), 100 μl ascorbic acid (100 mM) and 100 μl of homogenate. The reaction mixture was incubated in shaking water bath at 37 °C for 1 h, and then 500 μl trichloroacetic acid (10%) was added. To end, 500 μl thiobarbituric acid (0.67%) was added and all the tubes were placed in a water bath to boil for 10 min, and then transferred to crushed ice bath before centrifuging at 2500×g for 15 min. The quantity of MDA manufactured in each of the samples was evaluated by measuring the OD of the supernatant at 535 nm against a reagent blank. The outcomes were expressed as nM MDA/min/mg tissue at 37 °C using a molar extinction coefficient of 1.56 × 10^5^/M/cm.

##### Hydrogen peroxide assay

Estimation of hydrogen peroxide activity in tissue samples was monitored by method described earlier [48]. 100 μl of homogenate was amalgamated with reaction mixture comprising; 500 μl phosphate buffer (0.05 M, pH 7), 100 μl phenol red (0.28 nM), 250 μl dextrose (5.5 nM) and horse radish peroxidase (8.5 units) and incubation was done at room temperature for 60 min. 100 μl of NaOH (10 N) was added following centrifugation at 800×g for 5 min. The absorbance of the supernatant was checked at 610 nm against reagent blank. Production of H_2_O_2_ was measured as nM H_2_O_2_/min/mg tissue by using the standard curve of phenol red oxidized by H_2_O_2_.

##### Nitrite assay

For the execution of nitrite assay, Griess reagent was utilized [49]. Briefly, tissue samples (100 mg each) were de-proteinised in 100 μl solution comprising 5% ZnSO_4_ and 0.3 M NaOH, which were then centrifuged at 6400×g for 15–20 min and supernatant was collected. Griess reagent (1.0 ml) was added to the cuvette, and the spectrophotometer (Smart Spec TM Spectrophotometer) was blanked at wavelength of 540 nm, then the supernatant was added to the cuvette having Griess reagent. Standard curve of sodium nitrite was utilized for the quantification nitrite concentration in cardiac tissues.

#### Histopathological examination

Histopathological examination was performed for the assessment of cardiac injuries. Tissue samples from each group were fixed in a fixative containing absolute alcohol (85 ml), glacial acetic acid (5 ml) and 40% formaldehyde (10 ml). After dehydration, tissue samples were fixed in paraffin to prepare blocks for microtomy. Tissues were sectioned 4–5 μm with microtome and stained with hemotoxilin-eosin (H&E) and were examined under light-microscope-(DIALUX 20 EB) at 40X.

### Statistical analysis

Data are expressed mean ± SEM (*n* = 6). One way analysis of variance (ANOVA) followed by Tukey’s test was used for analyzing the statistical differences between different treatment groups using Graph pad prism 5 software. Level of significance was set at *p* < 0.05.

## Results

### Overall appearance, body weight and heart/body weight ratio

Rats were observed throughout the experiment for pain and distress by monitoring variations in food/water intake, mobility, and body weight. Animals looked healthy except the DOX group; in which rats appear feeble and their fur became scruffy. DOX did not cause significant decrease in heart weight/body weight (HW/BW) ratio, while significant decrease in body weight was noticed in rats treated with DOX alone or in combination with low dose AHE (200 mg/kg b.w dose) compared to control group. Maximum restoration was observed in DOX + AHE (400 mg/kg.b.w) and DOX + Sily (100 mg/ kg b.w) groups as against the DOX group (Table 2). No significant changes in the BW gain or HW/BW ratio were observed in rats treated with AHE alone. No mortalities were noticed in any of treatment groups.

**Table 2:** Effect of DOX and/or AHE treatment on body weight, heart weight and heart /body weight ratio of rats

Treatment (mg/kg)	Body weight (BW)(g)		Heart weight (HW)(g)	Ratio (HW/BWx10^3^)
	Initial	Final		
Control	219.0 ± 0.577	250.3 ± 0.333^b^	0.501 ± 0.023	2.00
DOX	221.3 ± 0.667	226.3 ± 0.661^a^	0.76 ± 0.021^a^	3.35
AHE alone	220.3 ± 0.667	248.3 ± 0.671^b^	0.509 ± 0.01^b^	2.04
DOX + AHE (200)	221.7 ± 0.882	232.2 ± 0.611^a,b^	0.651 ± 0.019^a^**^b^*^c^**	2.80
DOX + AHE (400)	220.3 ± 0.882	244.7 ± 0.882^a,b,c^	0.534 ± 0.017^b^	2.18
DOX + Sily	222.0 ± 0.577	243.3 ± 0.333^a,b^	0.541 ± 0.018^b^	2.22

Data expressed as mean ± SEM (*n* = 6)

^a^significant difference of final body weight of group Vs. Control group at *p* < 0.0001

^b^significant difference of final body weight of group Vs. DOX-treated group at *p* < 0.0001

^c^significant difference of final body weight of DOX + AHE (200 mg/kg) treated group Vs. DOX + AHE (400 mg/kg) treated group at *p* < 0.001

*, **indicate significance at *p* < 0.05 and *p* < 0.001. Non-significant difference (*p* > 0.05) was recorded between control and AHE alone treated group in all parameters. (One way ANOVA followed by Tukey’s multiple comparison tests). Sily-Silymarin

### Effect of AHE on serum cardiac injury biomarkers

**Fig. 1:**
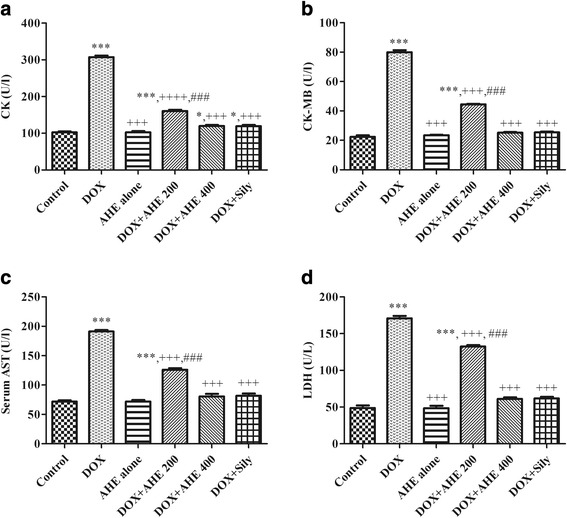
Effect of AHE treatment on serum markers of cardiac injury. **a** CK, (**b**) CK-MB, (**c**) AST, (**d**) LDH. Data are represented mean ± S.E.M (*n* = 6). Asterisks *, **, *** indicates *p* < 0.05, 0.001, 0.0001 vs. control; ^+, ++, +++^ indicates *p* < 0.05, 0.001, 0.0001 vs. DOX; ^#, ##, ###^ indicates *p* < 0.05, 0.001, 0.0001 vs. AHE (200 mg/kg b.w)

Figure 1 illustrates the serum cardiac marker enzyme levels in different treatment groups. Activities of serum markers; CK, CKMB, AST and LDH were significantly (*p* < 0.0001) higher in DOX inoculated group specifying myocardial injury. AHE ameliorated the toxic effect of DOX in a dose dependent manner and best protective effect was observed at AHE high dose. High dose of AHE showed comparable results as silymarin.

### Hematological findings

AHE (400 mg/kg) co-treated rats exhibited significant (*p* < 0.0001) improvement in most of the hematological parameters compared to DOX-treated rats (Table 3). These observations were confirmed by increased RBCs, Hb%, PCV, MCV, MCH, MCHC values and platelets counts. However, a significant (*P* < 0.01) reduction in RBCs and platelets counts as well as a significant (*P* < 0.01) increase in lymphocytes counts was recorded in DOX-treated rats compared to control rats.

**Table 3:** Effect of *A. hydaspica* AHE fraction on hematological parameters

Treatment (mg/kg)	RBC (10^6^/μl)	WBC (10^3^/μl)	Hb (g/Dl)	PCV (%)	MCV (fL)	MCH (pg)	MCHC (g/dL)	Neutrophil (10^3^/μl)	Lymphocytes (10^3^/μl)	Platelets (10^3^/mm^3^)
Control	8.01 ± 0.33	9.12 ± 0.51	13.13 ± 0.87	43.9 ± 1.22	73.11 ± 2.54	16.11 ± 1.11	29.57 ± 2.50	34.53 ± 2.71	22.11 ± 2.56	709 ± 3.22
DOX	4.61 ± 0.37^a^	5.11 ± 0.44^a^	10.08 ± 0.79^a^	22.65 ± 1.45^a^	59.98 ± 2.68^a^	10.20 ± 0.99^a^	19.89 ± 1.98^a^	21.10 ± 2.54^a^	47.81 ± 2.76^a^	386 ± 3.29^a^
AHE alone	8.10 ± 0.41^b^	9.19 ± 0.49^b^	13.23 ± 0.81^b^	45.11 ± 1.24^b^	74.01 ± 2.35^b^	16.55 ± 1.01^b^	29.9 ± 2.11^b^	35.11 ± 2.67^b^	21.21 ± 2.88^b^	714 ± 3.11^b^
DOX+ AHE (200)	6.01 ± 0.39^a^*,^c^**	6.67 ± 0.43^a,b,c^	11.01 ± 0.76^a^**^,b,c^	30.12 ± 1.56^a,b,c^	63.9 ± 2.60^a,c^*	12.87 ± 0.98^a,b^**^,c^*	22.34 ± 1.89^a,c^**	24.41 ± 2.71^a,c^**	39.65 ± 2.78^a^*^,b,c^**	498 ± 3.21^a,b,c^
DOX+ AHE (400)	7.95 ± 0.42^b^	8.98 ± 0.47^b^	12.80 ± 0.79^b^	39.50 ± 1.60^b^	68.99 ± 2.22^b^	14.98 ± 1.06^b^	27.65 ± 2.02^b^	30.22 ± 2.45^b^	27.32 ± 2.81^a^*^,b^	667 ± 3.78^a,b^
DOX+ Sily	7.96 ± 0.40^b^	8.97 ± 0.45^b^	12.88 ± 0.81^b^	39.23 ± 1.71^b^	68.56 ± 2.44^b^	15.08 ± 1.04^b^	27.9 ± 2.00^b^	31.01 ± 2.66^b^	27.50 ± 2.56^a^*^,b^	670 ± 3.66^a,b^

Data expressed as mean ± SEM (*n* = 6)

^a^significant difference Vs. Control group at *p* < 0.0001

^b^significant difference Vs. DOX-treated group at *p* < 0.0001

^c^significant difference of DOX + AHE (200 mg/kg) treated group Vs. DOX + AHE (400 mg/kg) treated group at *p* < 0.001

*, **indicate significance at p < 0.05 and *p* < 0.001. Non-significant difference (*p* > 0.05) was recorded between control and AHE alone treated group in all parameters. (One way ANOVA followed by Tukey’s multiple comparison tests). Sily-Silymarin

### Effect of AHE on cardiac antioxidant enzyme status

Antioxidant polyphenolic compounds are indispensable in the reclamation of reactive oxygen species (ROS) and help to maintain cellular equilibrium. In order to characterize the protective effect of AHE; alteration in antioxidant enzyme level were estimated after DOX treatment.

Table 4 demonstrates the protective effect of AHE on phase I antioxidant enzymes of heart i.e., CAT, POD, SOD and QR. In comparison to the control group, the level of CAT, POD, SOD and QR were significantly (*p* < 0.0001) decreased after the DOX inoculation. Co-administration of AHE with DOX restored the level of these enzymes in a dose dependent manner, and at high dose it was statistically similar to that of the silymarin treated group.

**Table 4:** Effect of Doxorubicin (DOX) and AHE treatment on cardiac tissue antioxidant enzymes

Group	POD (U/min)	SOD (U/mg protein)	CAT (U/min)	QR (nM/min/mg protein)
Control	10.66 ± 0.538^b^	1.146 ± 0.037^b^	15.76 ± 0.118^b^	137.5 ± 0.735^b^
DOX	5.970 ± 0.560^a^	0.7845 ± 0.027^a^	9.025 ± 0.090^a^	83.45 ± 0.416^a^
AHE alone	10.92 ± 0.531^b^	1.145 ± 0.049^b^	15.66 ± 0.197^b^	137.7 ± 0.392^b^
DOX + AHE (200)	8.63 ± 0.363^a,b^**	0.9799 ± 0.027^b^*	12.87 ± 0.297^a,b,d^	117.6 ± 0.652^a,b,d^
DOX + AHE (400)	9.600 ± 0.3464^b^	1.042 ± 0.025^b^**	14.95 ± 0.216^b,c^	131.0 ± 1.621^a^**^,b,c^
DOX + Sily	9.590 ± 0.3406^b^	1.055 ± 0.053^b^**	14.93 ± 0.256^b^	130.4 ± 0.7148^a,b^

Values expressed as mean ± SEM

^a^Significance at *p* < 0.0001 Vs. control group

^b^Significance at *p* < 0.0001 Vs. Doxorubicin (DOX) group

^c^Significance at *p* < 0.0001 of DOX + AHE 400 mg/kg group Vs. DOX + AHE 200 mg/kg group

^d^Significance at *p* < 0.0001 of AHE co-treatment groups Vs DOX + Sily group

*, **Significant difference at *p* < 0.05 and *p* < 0.001 respectively. Non-significant difference (*p* > 0.05) was recorded between control and AHE alone treated group in all parameters. (One way ANOVA followed by Tukey’s multiple comparison tests). Sily-Silymarin

Phase II antioxidant enzymes play a vital role in for detoxification of free radicals and synchronize action of different antioxidants is indispensable for maintaining redox balance. DOX-treatment showed significant (*p* < 0.0001) depletion of GSH, γ-GT, GR, GST, and GPx activity as compared to control animals (Table 5). DOX-induced deterioration in cardiac antioxidant enzyme levels was prevented noticeably with co-treatment of AHE in dose dependent manner. Activity level enzymes at high dose of AHE were statistically similar to the silymarin treated group. AHE alone treatment exhibited non-significant difference in the activity level of phase I and phase II antioxidant enzymes in comparison to control group.

**Table 5:** Effect of Doxorubicin (DOX) and different treatments of AHE on cardiac enzymatic antioxidant levels and GSH profile

Group	GSH (μM/g tissue)	GR (nM/min/mg protein)	GST (nM/min/mg protein)	γ-GT (nM/min/mg Protein)	GPx (nM/min/mg Protein)
Control	20.55 ± 0.280^b^	154.9 ± 0.96^b^	148.6 ± 0.665^b^	303.5 ± 0.811^b^	122.4 ± 0.639^b^
DOX	12.36 ± 0.490^a^	108.7 ± 1.095^a^	108.9 ± 1.105^a^	98.54 ± 1.106^a^	63.20 ± 1.027^a^
AHE alone	20.66 ± 0.237^b^	155.6 ± 0.439^b^	148.4 ± 0.815^b^	306.9 ± 0.401^b^	122.9 ± 0.285^b^
DOX + AHE (200)	14.37 ± 0.38^a,b^*^,d^	122.9 ± 1.14^a,b,d^	122.8 ± 0.984^a,b,d^	188.8 ± 0.095^a,b,d^	85.37 ± 0.540^a,b,d^
DOX + AHE (400)	18.59 ± 0.44^a^*^,b,^ ^c^	141.2 ± 1.00^a,b,c^	140.6 ± 0.723^a,b,c^	291.6 ± 0.69^a,b,c^	114.1 ± 0.845^a,b,c^
DOX + Sily	18.77 ± 0.360^b^	140.3 ± 1.55^a,b^	139.2 ± 0.4623^a,b^	289.0 ± 0.68^a,b^	115.9 ± 1.290^a^**^,b^

Values expressed as mean ± SEM

^a^Significance at *p* < 0.0001 Vs. control group

^b^Significance at *p* < 0.0001 Vs. Doxorubicin (DOX) group

^c^Significance at *p* < 0.0001 of DOX + AHE 400 mg/kg group Vs. DOX + AHE 200 mg/kg group

^d^Significance at *p* < 0.0001 of AHE co-treatment groups Vs DOX + Sily group

*, **Significant difference at *p* < 0.05 and *p* < 0.001 respectively. Non-significant difference (*p* > 0.05) was recorded between control and AHE alone treated group in all parameters. (One way ANOVA followed by Tukey’s multiple comparison tests). Sily-Silymarin

### Assessment of cardiac tissue protein content and oxidative stress biomarkers

Table 6 showed profile of proteins and oxidative stress marker i.e., TBARS, H2O2 and nitrite content of different groups. Cardiac tissue protein contents were reduced by DOX (*p* < 0.0001) in comparison to the control group. Animals administered with AHE (400 mg/kg) alone showed an increase in protein contents as compared to the AHE (200 mg/kg) + DOX, AHE (400 mg/kg) + DOX and only DOX group. DOX inoculation markedly increased cardiac tissue levels of MDA, H_2_O_2_ and nitrite contents compared to control group (*p* < 0.0001). Co-administration of AHE substantially prevented the rise in MDA level in a dose dependent manner, and non-significant difference was observed at higher dose in comparison to that of the control group. Level of H_2_O_2_ and nitrite content was dropped markedly and at the higher dose of AHE their level was statistically similar to that of the silymarin treated group. However, even at the higher dose of AHE their level was significantly higher against the control group. AHE, when treated in the absence of DOX, non-significant alteration in the level of above mention parameters as compared to control group.

**Table 6:** Effect of Doxorubicin (DOX) and different treatments of AHE on cardiac tissue protein, H_2_O_2_, nitrite content and lipid peroxidation

Group	Protein (μg/mg Tissue)	H_2_O_2_ (nM/min/mg Tissue)	Nitrite (content μM/ml)	TBARS (nM/min/mg protein)
Control	1.638 ± 0.033^b^	1.932 ± 0.015^b^	42.59 ± 0.552^b^	2.874 ± 0.180^b^
DOX	1.122 ± 0.0323^a^	5.854 ± 0.011^a^	78.12 ± 0.499^a^	7.575 ± 0.573^a^
AHE alone	1.614 ± 0.015^b^	1.911 ± 0.049^b^	41.72 ± 0.650^b^	2.859 ± 0.086^b^
DOX + AHE (200)	1.392 ± 0.036^a,b^	3.950 ± 0.003^a,b,d^	66.47 ± 1.456^a,b,d^	6.159 ± 0.091^a,b^*^,d^
DOX + AHE (400)	1.490 ± 0.018^a^*^,b^	2.719 ± 0.006^a,b,c^	49.94 ± 0.770^a,b,c^	3.258 ± 0.167^b,c^
DOX + Sily	1.509 ± 0.027^b^	2.645 ± 0.004^a,b^	50.60 ± 0.322^a,b^	3.233 ± 0.151^b^

Values expressed as mean ± SEM

^a^Significance at *p* < 0.0001 Vs. control group

^b^Significance at *p* < 0.0001 Vs. Doxorubicin (DOX) group

^c^Significance at *p* < 0.0001 of DOX + AHE 400 mg/kg group Vs. DOX + AHE 200 mg/kg group

^d^Significance at *p* < 0.0001 of AHE co-treatment groups Vs DOX + Sily group

*Significant difference at *p* < 0.05 and *p* < 0.001 respectively. Non-significant difference (*p* > 0.05) was recorded between control and AHE alone treated group in all parameters. (One way ANOVA followed by Tukey’s multiple comparison tests). Sily-Silymarin

### Histopathological examination of heart

**Fig. 2:**
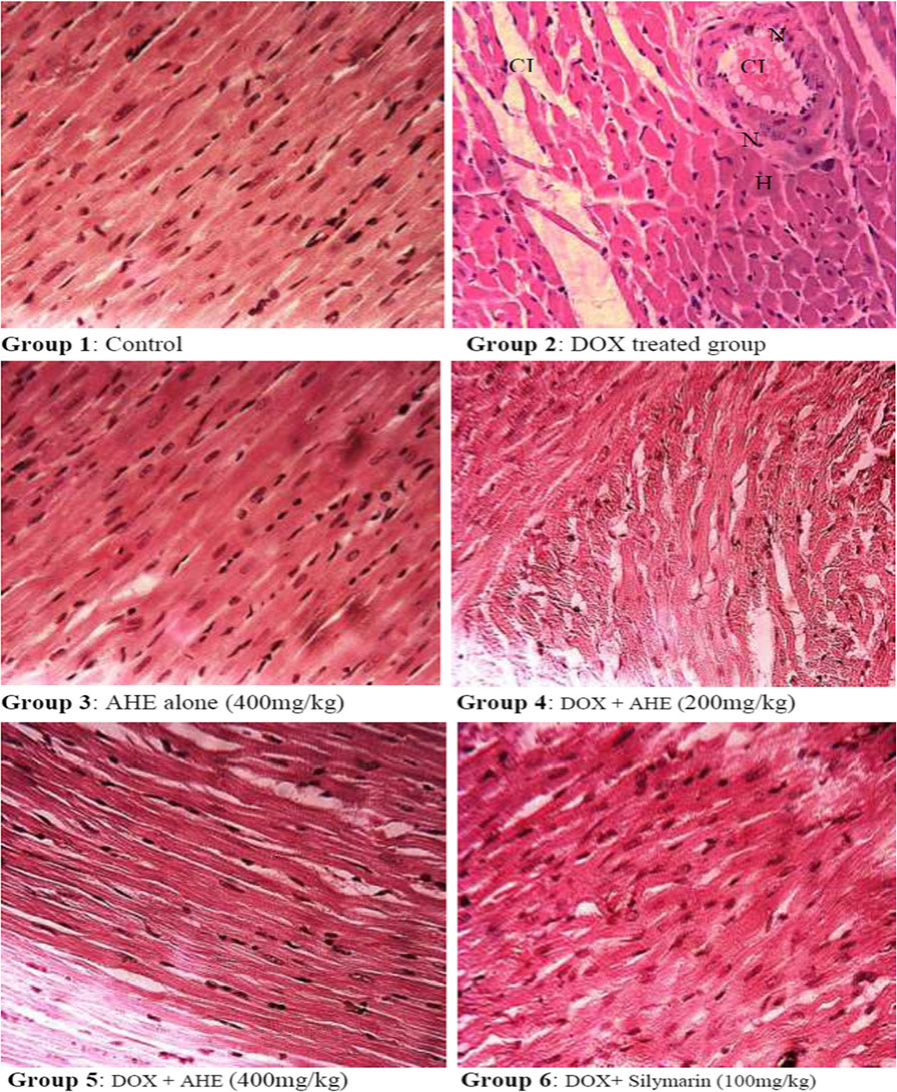
Histopathological changes induced by Doxorubicin and protective effect of AHE in rat heart (H&E staining, magnification 40X). Group 1: Cardiac section from control rats showing normal morphology. Group 2: cardiac sections from DOX-treated rats reveal degenerative changes. Group 3: Represents cardiac section from AHE alone treated rats. Group 4: AHE (200 mg/kg b.w) showed reduced degenerations. Group 5: AHE (400 mg/kg b.w) results in significant protection against DOX induced cardiac injury. Group 6: Showed protective effect of Silymarin treatment. AHE: *A.hydaspica* ethyl acetate fraction, DOX: Doxorubicin, H: hypertrophy, N: necrosis, CI: cellular infiltrations

The morphological examination of heart from different trial groups exhibited series of variations from no injury (control group) to mild lesions (AHE + DOX treated groups) to severe damage (DOX group). The control animals showed regular cardiomyocytes histoarchitecture with no visible signs of degenerations, fibrosis or necrosis (Fig. 2). DOX-intoxicated group showed exclusive pathological changes in cardiac tissue morphology showing inflammatory infiltrations, eosinophilic degeneration, necrosis in muscle fibers, hypertrophy of muscle fibers, distortion in blood capillaries, disturbance in the trabeculae of heart, retrogressive lacerations in muscle fibres, vacuolated muscle fibers and interstitial edema. Cardiac tissue sections from AHE (400 mg/kg b.w) + DOX group reveals myocardium of nearly normal appearance, significantly low occurrence of the degenerations and absence of necrosis, when compared with cardiac tissue sections from rats exposed only to DOX alone. However, AHE 200 mg/kg b.w co treatment group presented some incidence of morphological aberrations viz. irregular direction of cardiomyocytes, occasional interstitial edema, very mild inflammatory cell infiltrations and slight myocardium degenerations. However, the incidence of necrosis was still considerably lower than DOX group. Silymarin treatment showed normal cardiac muscle fibers with mild sign of toxicity in small restricted foci, less capillary dilatation and vacuolar changes in comparison to DOX-alone treated group, and most of the muscle fibers appear as control group indicating protective and comparable effects of AHE high dose treatment to silymarin.

## Discussion

Doxorubicin (DOX) therapy is associated with irreversible and progressive cardiomyopathy that restricted its clinical implication. Oxidative stress has been ascribed as the dominant cause in DOX-induced cardio-toxicity. Failure of antioxidant defense of cardio-myocytes marks the heart a major target for DOX induced injuriousness [50]. In the literature, it has been studied that antioxidant treatment provides protection against DOX mediated cardiotoxicity [51, 52]. In view of the fact that medicinal plants are being tested for their possible therapeutic potential against ROS induced damages due to their antioxidant potential, the current study was conducted to evaluate the protective potential of polyphenol rich AHE extract against DOX induced myocardial injury.

Detection of cardiac insult, tissue ischemia and myocardial infarction employs estimation of recognized cardiac marker enzymes i.e., Creatine kinase (CK), cardiac creatine kinase MB fraction (CK-MB), AST, ALT, ALP, LDH and cholesterol present in the serum [53, 54]. All of the exemplified parameters are not only demarcated to cardiac tissue except CK and CKMB, their enhanced levels in serum may possibly an indication of non-cardiac tissue damages for instance; liver injury. CKMB appear to be the most sensitive and specific markers of myocardial injury, and WHO acknowledged it as a gold standard signal of myocardial impairment [55]. The cardiac cell membrane integrity loss as a result of free radical mediated lipid peroxidation causing leakage of enzymes [51]. This accounts for the diminished activities of these enzymes in cardiac tissue as these enzymes released into the blood, thus increasing their levels in the serum as a signal of myocyte damage [56, 57].

DOX induced excessive elevation of CK, CKMB and LDH concentration in the serum as compared to control is an indicator of myocardial injury. Our outcomes were consistent with the observations of EL-Sayed and colleagues demonstrating that DOX treatment predominantly elevated serum CKMB, CK and LDH activity; the most sensitive biomarkers of myocardial cell injury [58]. The mechanism involves in the release of these marker enzymes seems to be oxidative damage to heart tissue and consequent release of its insides substances into the circulation. Enhanced production of free radicals, especially superoxide anion radicals could enhance the inflammatory cascade in the cell wall and might result in atrial endothelial dysfunction. Beside these ventricular alterations, continuing myocyte degeneration and abridged coronary reserve might be the causes of enzyme leakage [59]. Normalization of the serum content of CK, CK-MB, AST and LDH in experimental groups treated with AHE co-treatment group’s shows improved cardiac function in DOX intoxicated groups hence, indicates the cardio-protective potential of *A. hydaspica*. The results were comparable with standard Silymarin. Presence of gallic acid in AHE might attribute to the protective effect as previous studies indicated that gallic acid markedly brought down serum cardiac and lipid biomarkers in DOX treated rats [60]. Our outcomes are similar to previous findings on protective effect of plant extracts against DOX induced cardiotoxicity [58]. The current serological findings have an outstanding relationship with histological investigation of cardiac tissue of rat.

Hematopoietic system is a rational indicator to delineate the physical condition. Measurement of cellular component of blood is important in establishing the body’s functional status as a result of exposure to toxicants [61]. The present study indicated that co-treatment with AHE had effectively ameliorated the hematological anomalies induced by DOX in a dose dependent manner. The deleterious influence of DOX on blood parameters was verified by the significant drop in RBCs, Hb and platelets counts with consequent drop in the values of MCV, MCH, MCHC and PCV. DOX intoxication might lead to anemia as a result of either altered activity of hematopoietic tissues, impaired erythropoiesis, and/or defective iron metabolism [62]. Furthermore chronic administration of DOX induced a reduction in the number of platelets and an increase in the number lymphocytes in the blood of rats. Previous studies also confirm doxorubicin-mediated thrombocytopenia via cytotoxic effect of DOX on platelets. DOX induced bone marrow toxicity has been suggested as underlying cause of reduced platelet counts. Furthermore reactive oxygen species (ROS) generation, decreased glutathione levels and consequent protein thiol depletion were exposed to be the cause of the DOX-persuaded platelet cytotoxicity [63]. However, the increase in the lymphocytes number might be the result of inflammation during DOX treatment. AHE was found to have beneficial effects against DOX-persuaded deterioration in most of hematological parameters and RBCs indices as it increased number of RBCs and Hb concentration and other hematological parameters about to normal levels. Our results are in line with previous studies indicating the ameliorating potential of *Acacia species* on an increase in erythropoiesis and other hematological parameters due to their immune-stimulatory properties [64, 65]. The ameliorative effect of AHE might be due to reduction of lipid peroxidation level with subsequent prevention of free radicals induced damage through its antioxidant activity achieved by its active compounds.

Antioxidant enzymes, i.e. SOD, POD, CAT, QR, GSH, GPx, γ GT, GST provides defense against oxidative stress mediated tissue injury. GSH in cellular defense system has the conjugating aptitude with free radicals and metabolites, consequently alleviating the membranes from the damaging influences of lipid peroxides. Previous study demonstrated that cellular GSH decrease is directly linked with LPO caused by lethal agents [66]. The rise in MDA level (LPO product) could be ascribed to DOX persuaded production of oxygen free radicals that arouse widespread tissue mutilations, countering with membrane proteins, lipids and nucleic acids [67, 68]. The deterioration in GSH level in DOX-treated rats ensued LPO augmentation thus confirming cardiotoxicity [69]. The outcomes of the current study are in harmony with earlier findings, illustrating that myocardial antioxidant protection system was functioning at a lower rate apart from the higher level of oxidative stress in DOX intoxicated cardiotoxicity [51, 70]. In the current investigation, the noticeable decrease in cardiac tissue antioxidant enzyme levels and increase in oxidative stress markers (MDA, NO and H_2_O_2_) besides decreased in cardiac tissue protein content are confirmations of the oxidative stress instigated by DOX treatment. It was observed that AHE high dose co-treatment was able to antagonize DOX induced diminution of antioxidant enzymes, and reverse the levels near normal, and its efficacy was comparable to the silymarin treated group. Analogous to current outcomes, previous research reported that silymarin treatment before ischemic-reperfusion-prompted myocardial infarction maintained the cardiac marker enzymes compared with isoproterenol-administered rats [71], similar protective effect of silymarin against CP induced cardiotoxicity was also reported previously [51]. The protective potential of AHE attributes to the presence of phenolic and flavonoid compounds in AHE [26]. As gallic acid and catechin are major polyphenols in AHE and previous studies on gallic acid affirms that its mechanism of protection is through the restoration of the endogenous antioxidant system by scavenging the reactive oxygen species [60]. A similar study on catechin proves that It protect DOX induced cardiotoxicity in rats in a dose dependent manner [72]. Catechin has anti-inflammatory and anti-oxidative effects against Adriamycin induced cardiotoxicity, which results from intense cardiac oxidative stress and inflammation [73]. The most common mechanism of action for almost all phenolic compounds is inhibition of oxidative stress by scavenging free radicals and enhancing cellular antioxidant defense mechanisms in cardiomyocytes [74, 75]. In current finding, the obvious increase in tissue antioxidant status, decrease in lipid peroxidation and other oxidative stress markers suggest that protection afforded by AHE may be mediated through the modulation of cellular antioxidant enzyme levels and by averting the generation of free radicals in myocardial cells.

**Fig. 3:**
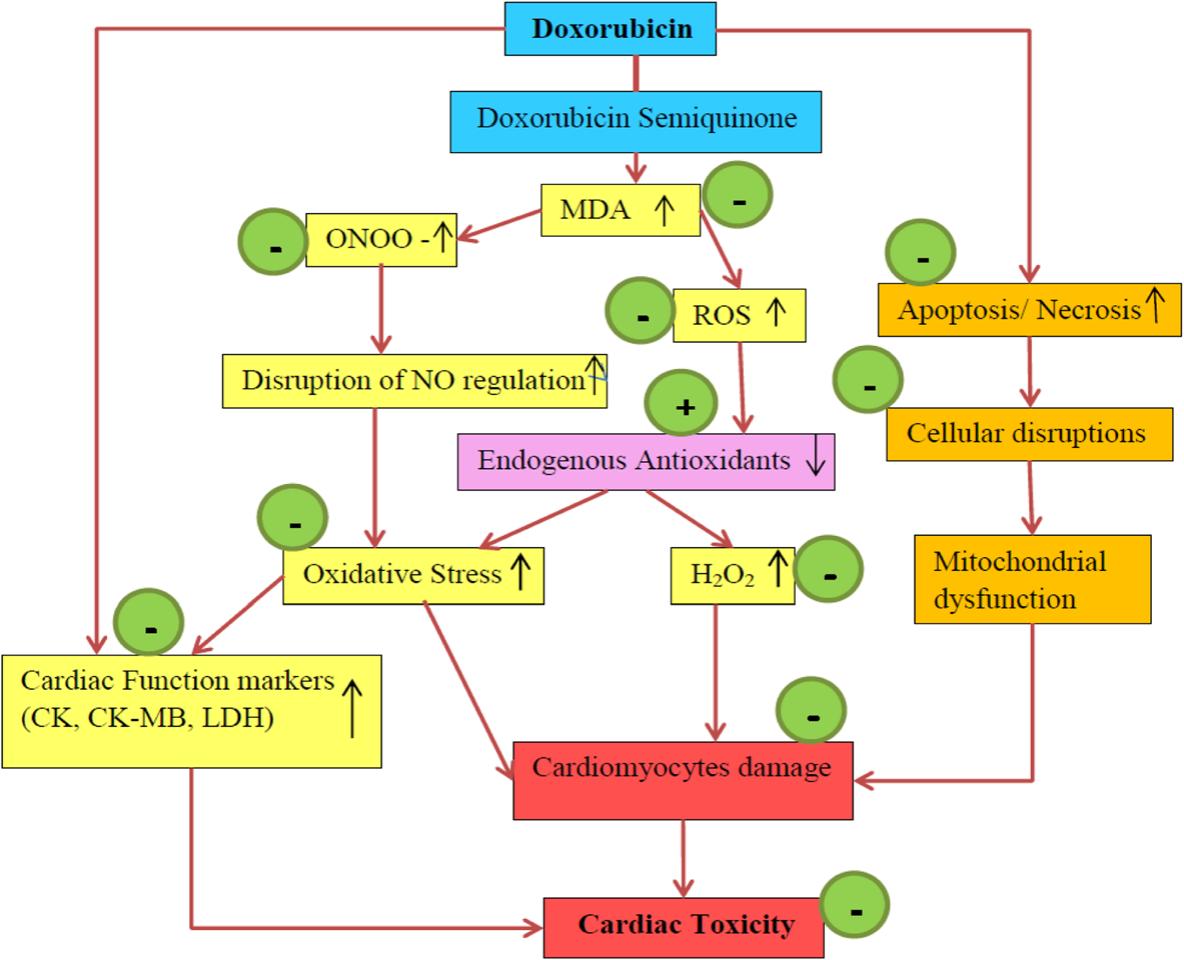
Hypothetical pathway describing the possible underlying mechanism of DOX induced cardiotoxicity and protective effect of AHE. Green circles indicate points of AHE treatment effect, − sign indicates inhibition and + sign indicates augmentation

Histopathology showed myocardial atrophy, nuclear condensation of chromosomes and cytoplasmic vacuoles in DOX intoxicated cardiac tissues. AHE in a dose depend manner demonstrated considerable prevention in the structural changes in cardiomyocytes of DOX-intoxicated animals. DOX treatment induced noticeable fatty obstruction in the blood vessel and clearance of cytoplasm with foamy appearance while nuclear deterioration was also seen in some areas. All these morphological deteriorations caused by DOX were considerably reverted by AHE co-treatments. The protective effect of AHE is comparable to silymarin treatment. Analogous findings were demonstrated by the previous study illustrating that silymarin suppressed the adriamycin induced cardiotoxicity in male albino rats [76]. The protective effect of AHE might be due to its antioxidant potential, AHE protective cardiomyocytes damages by augmenting tissue antioxidant status and inhibiting oxidative stress mediated free radical generation. Figure 3 summarizes the possible mechanism of AHE protective effect against DOX mediated cardiotoxicity. Previous studies have also authenticates the beneficial effect of antioxidant in doxorubicin or adriamycin induced myocardial damage [77, 78]. Du and Lou, 2008 illustrated that the cardiovascular protective effects of grape seed against DOX induce toxicity is believed to be ascribed to its antioxidant properties. Grape seed polyphenols, catechin and proanthocyanidin B4 (PC B_4_) pretreatment would protect cardiomyocytes against doxorubicin-induced toxicity by decreasing ROS generation, preventing DNA damage, regulating the expression and signaling pathway of pro-apoptotic and anti-apoptotic proteins [79]. Similarly Singh and his coworkers reported the cardio-protective effect of butanol fraction of *Terminalia arjuna* bark against DOX induced morphological alteration by alleviating morphological changes and decreasing oxidative stress [80]. The observed protective effect may be accredited to the distinct or synergistic effect of bioactive phytochemicals present in the AHE fraction.

## Conclusion

The implication of oxidative stress in the etiology of chemotherapeutic drug induced cardiovascular ailments suggests that medicinal plants possessing antioxidant potential represents a promising avenue for treatment. Strategies for the intervention and prevention of cardiovascular illnesses require an understanding of the basic mechanism by which the prophylactic agents may potentially prevent the toxic effects. The current study used chronic model of DOX cardiotoxicity in trial experiment and the results shows AHE may be beneficial for DOX-induced cardiotoxicity by ameliorating oxidative stress. However, this basic research needs to be further confirmed in a more clinically relevant model to explicate the mechanism and develop strategies in prevention against DOX-induced cardiotoxicity.

## Abbreviations

AHE: *Acacia hydaspica* ethyl-acetate extract
AST: Aspartate aminotransferase
CAT: Catalase
CK: Creatine kinase
CK-MB: Cardiac creatine kinase MB fraction
DOX: Doxorubicin
GPx: Glutathione peroxidase
GSH: Reduced glutathione
GST: Glutathione s transferase
H_2_O_2_: Hydrogen peroxide
Hb: Hemoglobin
LDH: Lactate dehydrogenase
LPO: Lipid peroxidation
MCH: Mean corpuscle hemoglobin
MCHC: Mean corpuscle hemoglobin concentration
MCV: Mean corpuscle volume
MDA: Malondialdehyde
NO: Nitric oxide
PCV: Packed cell volume
POD: Peroxidase
QR: Quinone reductase
RBCs: Red blood cells
ROS: Reactive oxygen species
SOD: Superoxide dismutase
γ GT: γ-Glutamyl transpeptidase
